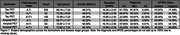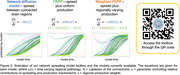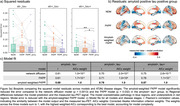# Demonstration of an open‐source toolbox for network spreading models: regional amyloid burden promotes tau production in Alzheimer's disease

**DOI:** 10.1002/alz.093791

**Published:** 2025-01-09

**Authors:** Elinor Thompson, Anna Schroder, Tiantian He, Antoine Legouhy, Xin Zhao, James H. Cole, Neil P Oxtoby, Daniel C Alexander

**Affiliations:** ^1^ UCL Centre for Medical Image Computing, Department of Computer Science, University College London, London United Kingdom; ^2^ UCL Centre for Medical Image Computing, Department of Medical Physics and Biomedical Engineering, University College London, London United Kingdom; ^3^ University College London, London, London United Kingdom

## Abstract

**Background:**

Connectome‐based models of disease propagation are used to probe mechanisms of pathology spread in neurodegenerative disease. We present our network spreading model toolbox that allows the user to compare model fits across different models and parameters. We apply the toolbox to assess whether local amyloid levels affect production of pathological tau.

**Methods:**

Data: Regional tau‐PET and amyloid‐PET SUVRs from the Alzheimer’s Disease Neuroimaging Initiative (ADNI) study were downloaded in September 2023 (demographics in Figure 1). Subjects were separated into groups based on AT disease stage: 1) amyloid‐negative, tau‐negative; 2) amyloid‐positive, tau‐negative; 3) amyloid‐positive, tau‐positive. Positivity thresholds were defined using Gaussian mixture modelling. Data were averaged within groups to produce stage‐specific maps. Spreading models: We compared three models: the network diffusion model, Fisher‐Kolmogorov‐Petrovsky‐Piscounov (FKPP) and weighted‐FKPP models (Figure 2). Previously we showed that weighting the production rate of FKPP with regional vectors improves the model fit by accounting for different regional characteristics (He et al, MICCAI 2023). Here, we demonstrate this new “weighted‐FKPP” model, using regional amyloid as the fixed production weight. Models were initialised with unity pathology in a bilateral seed region, zero elsewhere. Parameter optimisation: Free parameters were fitted using Gaussian processes optimisation (α and seed region). NDM toolbox: All models are available in our toolbox: https://github.com/ethompson93/network_spreading_models. Null Modelling: we repeated the weighted‐FKPP model optimisation with a distribution of 100 randomly shuffled amyloid maps to demonstrate the spatial significance of the amyloid weighting.

**Result:**

Adding amyloid weighting to the production term in the FKPP model improved the model fit to the data for each group. The reduction in squared model residuals with the weighted FKPP model was significant only in the amyloid‐positive tau‐positive group (Figure 3). For all groups, the results from the FKPP weighted with amyloid had significantly improved model fit (Pearson’s R) compared to the null model (all p‐values<1×10‐10).

**Conclusion:**

Using our toolbox, we found that incorporating regional amyloid levels improves pathology‐spreading model fit to measured tau‐PET data, particularly at later stages of AD. This supports the hypothesis that amyloid promotes tau aggregation.